# Dose- and time-resolved immune states in cervical cancer radiotherapy: dynamic multimodal biomarkers for adaptive immunoradiotherapy

**DOI:** 10.3389/fimmu.2026.1981103

**Published:** 2026-10-22

**Authors:** Chengjian Xiao, Feilong Tian, Minli Kang, Fangping Xie

**Affiliations:** 1 Department of Radiation Oncology, Ganzhou Cancer Hospital, Ganzhou, China; 2 Department of Pathology, Ganzhou Cancer Hospital, Ganzhou, China

**Keywords:** dynamic biomarkers, image−guided adaptive brachytherapy, immunoradiotherapy, locally advanced cervical cancer, radiation−induced immune remodeling

## Abstract

Concurrent and maintenance immune checkpoint blockade improves survival for select high-risk locally advanced cervical cancer patients treated with definitive chemoradiotherapy, yet population-level efficacy cannot identify responders, optimal timing of benefit, or actionable biological targets for resistance. Fractionated pelvic radiotherapy induces dynamic immune perturbation: antigen release and interferon signaling occur alongside lymphocyte depletion, impaired antigen presentation, suppressive myeloid remodeling and resistant tumor clone selection. Pretreatment biomarkers fail to capture this evolving biological landscape. This review synthesizes evidence for dose- and time-resolved tissue/blood immunity, longitudinal MRI/PET imaging, delta radiomics, multimodal artificial intelligence, and HPV circulating tumor DNA. Two key conclusions are drawn. First, serial studies show heterogeneous immune remodeling modulated by radiation dose, sampling site, assay platform and time point. Second, mid-treatment tumor regression has reproducible prognostic evidence, and post-treatment viral circulating tumor DNA has prospective validation for residual-risk assessment and monitoring. We categorize biomarker functions as response assessment, prognosis, treatment-effect prediction and monitoring, define six evidence gates, and propose trial-restricted decision windows to guide future translational investigations.

## Introduction

Cervical cancer remains a major global cause of cancer incidence and mortality among women despite the preventability of most human papillomavirus (HPV)-associated disease and the expansion of vaccination and screening (1, 2). For patients with locally advanced cervical cancer (LACC), definitive external-beam radiotherapy with concurrent cisplatin followed by image-guided adaptive brachytherapy (IGABT) remains the curative backbone. Contemporary European guidelines emphasize adequate external-beam target coverage, timely brachytherapy, and completion of the overall treatment course because radiotherapy quality is itself a determinant of tumor control (3). In the prospective, multicenter EMBRACE-I cohort, MRI-based IGABT achieved 92% actuarial five-year local control among 1, 341 evaluable patients, establishing a treatment-quality benchmark for future biomarker studies (4). EMBRACE-II subsequently formalized advanced external-beam and brachytherapy planning aims while embedding translational imaging and tissue research (5). A biomarker-guided strategy that compromises IGABT, delays treatment, or mistakes inadequate dose delivery for biological resistance would not be precision medicine.

The phase 3 ENGOT-cx11/GOG-3047/KEYNOTE-A18 trial established a new therapeutic context for patients with newly diagnosed, high-risk LACC. Pembrolizumab given concurrently with chemoradiotherapy and continued as maintenance improved progression-free survival and, with longer follow-up, overall survival (6, 7). Yet the phase 3 CALLA trial, in which durvalumab was also delivered during and after chemoradiotherapy, did not meet its primary progression-free survival endpoint (8). Differences in agents, eligibility, radiotherapy, timing, duration, statistical maturity, and tumor biology may all contribute, but none has been proven to explain the divergent results. Together, these trials create a more precise question than whether immunotherapy “works” with pelvic chemoradiotherapy: which baseline and on-treatment states identify sensitivity, primary resistance, emerging resistance, or avoidable toxicity, and can those states be measured early enough to alter management? Most proposed cervical cancer biomarkers are static. Pretreatment stage, nodal status, tumor volume, programmed death-ligand 1 (PD-L1), tumor-infiltrating lymphocytes, gene-expression signatures, and radiomic phenotypes can stratify outcome, but they do not directly measure how a tumor-host system responds to fractionated radiation, cisplatin, brachytherapy, and immune checkpoint blockade. BIOEMBRACE illustrates both the value and the limit of this approach: in 264 patients treated within an MRI-guided brachytherapy platform, PD-L1, L1CAM, and MRI necrosis were associated with response or disease control, yet they were not tested for differential benefit from checkpoint blockade (9). The distinction between prognosis and treatment-effect prediction determines the study design required for each claim (10).

Longitudinal measurements offer a different opportunity. During chemoradiotherapy, serial magnetic resonance imaging (MRI) can quantify tumor shrinkage and evolving texture; repeated blood sampling can measure treatment-related lymphocyte loss, T-cell receptor dynamics, cytokines, and HPV circulating tumor DNA (ctDNA); and scheduled cervical sampling can reveal local interferon signaling, antigen-presentation programs, checkpoint expression, and immune-cell trafficking. In this Review, a dynamic biomarker is a measurement whose level or change is interpreted at a prespecified landmark; an immune state is a multicomponent configuration observed at one landmark; a trajectory requires change across at least two landmarks; and a decision window is a time at which a result could be available before a prespecified trial action. These signals occupy different biological scales, and their discordance can be informative. Local tumor activation may not be mirrored in blood, while peripheral lymphopenia may reflect host reserve, marrow exposure, systemic inflammation, and the dose delivered to circulating immune cells. Multimodal integration is therefore attractive only when modalities are aligned to the same biological time, evidentiary function, and clinical decision. Dose-resolved evidence records delivered dose at sampling, whereas time-resolved evidence records elapsed days from treatment initiation or completion.

Recent reviews have separately synthesized the immune effects of cervical-cancer treatment, PD-1/PD-L1 combinations, current immunotherapy trials, radiotherapy-driven immune reprogramming, spatial macrophage–T-cell ecology, and radiomics or artificial intelligence (AI) in precision radiotherapy (11–16). A 2026 review additionally framed gut and vaginal microbiomes as candidate modifiers of chemoradiotherapy response, toxicity, mucosal integrity, and host immunity, while emphasizing the need for longitudinal clinical validation (17). A recent temporal synthesis also organizes immune remodeling by treatment phase and proposes a biomarker framework (18). The contribution of the present Review is therefore not longitudinal sampling or multimodal coverage alone, but their integration around four biomarker functions, claim-specific evidence requirements, and treatment-by-biomarker interaction before treatment selection. This Review advances a specific argument: the next useful biomarker for cervical immunoradiotherapy is unlikely to be another pretreatment risk score. It should be a time-resolved system that distinguishes radiation-induced immune activation from suppression, quantifies residual disease, and estimates treatment-specific benefit before an actionable window closes. We synthesize the clinical and translational evidence for this framework, identify where current artificial-intelligence models overreach their evidence, and propose a prospective roadmap for adaptive trials.

This narrative review draws on a reference set assembled by October 2026. Search concepts combined cervical cancer with chemoradiotherapy or radiotherapy and longitudinal immune measurements, dynamic MRI or delta radiomics, circulating tumor or HPV DNA, and checkpoint biomarkers or treatment interaction. We used four operational categories: response, prognosis, treatment-effect prediction, and monitoring. We applied: biological plausibility; analytical validity; clinical association in the intended population and landmark; locked external validation; a prespecified treatment-by-biomarker interaction; and demonstrated clinical utility of the biomarker-triggered action. For imaging and AI studies, we additionally examined patient- and center-level separation, external validation, calibration, decision-curve analysis, temporal alignment, missing modalities, and whether the claimed action was tested. The synthesis draws on the prognostic/predictive distinction and FDA-NIH BEST terminology (10, 19); imaging and model appraisal follow current prediction-model, medical-imaging AI, and radiomics-readiness frameworks (20–23). For dynamic markers, randomization for the treatment decision should occur after marker measurement at the decision landmark; analyses of markers measured after initial randomization require a prespecified causal estimand and methods addressing post-randomization selection (24).

## The issue of defining biomarkers for clinical immunotherapy-radiotherapy

KEYNOTE-A18 enrolled 1, 060 patients with FIGO 2014 stage IB2-IIB node-positive disease or stage III-IVA disease. Participants received five cycles of pembrolizumab or placebo during chemoradiotherapy followed by 15 maintenance cycles. At the first analysis, 24-month progression-free survival was 68% versus 57% (hazard ratio [HR] for progression or death, 0.70; 95% confidence interval [CI], 0.55-0.89) (6). At a median follow-up of 29.9 months, 36-month overall survival was 82.6% with pembrolizumab and 74.8% with placebo (HR for death, 0.67; 95% CI, 0.50-0.90). Grade 3 or higher adverse events occurred in 78% and 70%, respectively, and potentially immune-mediated events in 39% and 17% (7). These results establish a clinically meaningful overall treatment effect for the trial-defined high-risk population, but the published aggregate estimates do not reveal whether benefit is uniform or concentrated in biological subgroups.

CALLA randomized 770 patients to durvalumab or placebo during and after chemoradiotherapy. The primary progression-free survival endpoint was not met (HR, 0.84; 95% CI, 0.65-1.08; P = 0.17) (8). Smaller trials provide biological granularity that phase 3 efficacy trials rarely capture (**Table 1**). In GOG-9929, circulating T-cell ICOS and PD-1 expression increased after chemoradiotherapy and was sustained or increased during subsequent ipilimumab; central and effector memory populations expanded after combined treatment, and greater baseline-to-post-CRT increases in TNFα, IL-6, and IL-8 were associated with inferior progression-free survival (25). A randomized phase 2 study of pembrolizumab during versus after pelvic chemoradiotherapy showed that both schedules were feasible in 94 evaluable patients, but neither progression-free nor overall survival differed between timing groups; planned translational analyses are therefore more informative for sequencing than the underpowered clinical comparison (26).

**Table 1:** Clinical and translational immunoradiotherapy evidence in locally advanced cervical cancer.

Study and design	Checkpoint schedule	Principal result	Biomarker implication and limitation
KEYNOTE-A18; phase 3; n=1, 060 (7)	Pembrolizumab during chemoradiotherapy, then maintenance	36-month overall survival 82.6% vs 74.8%; HR 0.67 (95% CI 0.50–0.90)	Establishes population-level benefit in trial-defined high-risk LACC; published aggregate data do not define an individual treatment-predictive marker.
CALLA; phase 3; n=770 (8)	Durvalumab during and after chemoradiotherapy	Progression-free survival HR 0.84 (95% CI 0.65–1.08); P = 0.17	Negative all-comer result; contrast with KEYNOTE-A18 generates hypotheses about agent, population and timing but does not prove the cause.
NRG-GY017; randomized phase 1; 40 randomized, 25 primary-endpoint evaluable (27)	Neoadjuvant plus concurrent atezolizumab vs concurrent atezolizumab	Greater early expansion of tumor-associated T-cell receptor clones; sensitivity analysis not significant; 2-year disease-free survival difference not significant	Shows that sequencing changes immune kinetics; too small for comparative efficacy or a treatment-selection rule.
Pembrolizumab timing study; randomized phase 2; n=94 evaluable (26)	Concurrent vs sequential pembrolizumab; no maintenance	Both schedules feasible; no progression-free or overall survival difference between timing arms	Safety evidence; translational endpoints are needed to explain timing rather than relying on underpowered survival comparisons.
GOG-9929; phase 1 translational cohort (25)	Ipilimumab after definitive chemoradiotherapy	Post-CRT ICOS/PD-1 increases persisted or rose during ipilimumab; memory expansion followed combined treatment; greater baseline-to-post-CRT increases in TNFα/IL-6/IL-8 correlated with poorer PFS	Supports serial host immune monitoring; outcome associations are exploratory.
COLIBRI; phase 2 window trial; n=40 (28)	One cycle nivolumab plus ipilimumab before chemoradiotherapy, then maintenance nivolumab	CD8/FOXP3 ratio and 27-gene immune HOT score increased after induction	Biological priming signal in a single-arm design; does not isolate the contribution of induction or dual blockade.
Exploratory randomized phase 2; n=18 (29)	Chemoradiotherapy plus immunotherapy vs chemoradiotherapy	Residual tumor volume 3.0% vs 7.4%, P = 0.080; single-cell analysis of 12 pre- and on-treatment tumor samples from six patients showed pro-inflammatory remodeling	Mechanistically informative but severely underpowered; hypothesis-generating only.

CI, confidence interval; HR, hazard ratio; LACC, locally advanced cervical cancer. KEYNOTE-A18 and CALLA are separate placebo-controlled trials, not a head-to-head comparison of PD-1 and PD-L1 inhibition.

NRG-GY017 directly tested timing. Atezolizumab was given neoadjuvantly and concurrently or concurrently alone in node-positive LACC. Among 25 patients evaluable for the primary immune endpoint, the neoadjuvant arm showed greater peripheral expansion of tumor-associated T-cell receptor clones after the first cycle; the prespecified two-sample test was borderline, and a sensitivity analysis was not significant. Two-year disease-free survival was numerically 76% versus 56% but was not statistically different (27). This is not efficacy proof. It is evidence that moving checkpoint blockade before substantial radiation exposure can change the measurable immune trajectory. The COLIBRI window-of-opportunity trial further supports a priming concept. Forty patients received one cycle of nivolumab plus ipilimumab before chemoradiotherapy, followed by maintenance nivolumab. The CD8-to-FOXP3 cell ratio and a 27-gene immune “HOT” score increased after induction; baseline and induced immune activity were associated with complete response at treatment completion (28). Conversely, a randomized single-center phase 2 study reported in 2026 included only 18 patients. The addition of immunotherapy produced a numerical reduction in residual tumor volume that did not reach conventional significance, while single-cell RNA sequencing of 12 tumor samples from six patients (three per treatment group), collected one week before and two weeks after chemoradiotherapy initiation, showed increased antigen-presentation and chemotaxis programs, fewer regulatory T-cell niches and exhausted CD8 T cells, and a macrophage shift toward inflammatory states (29).

## Fractionated radiotherapy reshapes the cervical immune ecosystem

HPV-positive cervical cancer provides a biologically plausible setting for immunoradiotherapy. Viral E6 and E7 oncoproteins, recurrent genomic alterations, and heterogeneous antigen-presentation and immune-evasion programs create targetable immune recognition but also strong selection pressure for escape. The Cancer Genome Atlas described molecular subgroups with distinct genomic, epigenetic, and immune features rather than a single cervical cancer state (30). Pan-cancer immune profiling likewise demonstrates that immune composition and interferon activity vary markedly across and within tumor types (31). Spatially resolved studies reinforce that a biopsy-average immune score can conceal distinct ecological niches. Single-nucleus RNA sequencing with spatial transcriptomics in cervical squamous cell carcinoma identified metabolically distinct immune distributions and a myofibroblast program associated with restricted lymphocyte infiltration; immunohistochemistry and multiplex immunofluorescence supported the spatial observations (32). A separate spatial analysis mapped proliferative, invasive-edge, stress-adaptive, and differentiated epithelial programs shaped by extracellular-matrix signaling (33).

Definitive chemoradiotherapy, preoperative irradiation followed by surgery, short-course radiation, and recurrent/metastatic systemic therapy involve different populations and treatment contexts. Evidence from the latter settings is indirect for an on-treatment marker during definitive EBRT and brachytherapy and requires validation in the intended LACC pathway. **Table 2** identifies the reported population and dose or time landmark; **Table 3** summarizes the biomarker functions and validation required for each claim. Radiation can amplify recognition through DNA damage, antigen release, type I interferon signaling, and recruitment of antigen-presenting and effector cells. Preclinical studies show that tumor-cell irradiation can synergize with PD-L1 blockade and that fractionation influences systemic immune effects (34, 35). Cytosolic DNA sensing through cGAS-STING promotes type I interferon-dependent antitumor immunity, whereas radiation-induced TREX1 can degrade cytosolic DNA and limit immunogenicity at high per-fraction doses (36, 37). Immunogenic cell death is therefore not a binary property of radiation; its detection requires evidence of defined danger signals and immune consequences rather than the assumption that any tumor shrinkage is immunogenic (38).

**Table 2:** Representative dynamic biomarker studies and their highest supported evidence function.

Study/layer	Design and landmark	Signal	Supported function/evidence domains
Baseline tissue PD-L1 in BIOEMBRACE (9)	n=264 overall; PD-L1 evaluable n=223; pretreatment tissue; multicenter definitive CRT with MRI-guided brachytherapy; static comparator	PD-L1 >1% associated with inferior 5-year local control	G3: prognostic association under CRT/IGABT. Independent intended-use validation (G4) remains unestablished. This static comparator differs from dose-anchored PD-L1 induction.
Serial cervical cytobrushing (40)	n=20; baseline, weeks 1, 3, 5; definitive CRT; time-resolved	Early CD4/CD8 decline, later heterogeneity; local and blood changes diverged	G1: biological plausibility and serial immune change. Outcome-linked association (G3) and locked external validation (G4) remain unestablished.
Paired biopsy at 20 Gy (49)	n=30; baseline and 20 Gy; concurrent chemoradiotherapy; dose-resolved	Heterogeneous CD8/PD-L1 change; IRF1-linked activation in a subset	G3: exploratory short-term response association with CD8 change. Full analytical validation (G2) and locked external validation (G4) remain unestablished.
Dose-anchored PD-L1 study (53)	n=40; 10–14 and 18–22 Gy; serial sampling during radiotherapy; concurrent chemotherapy varied	PD-L1/NF-κB induction with CD8 and macrophage infiltration	G1: dose-anchored PD-L1 induction. Analytical and outcome-linked clinical validation (G2/G3), followed by intended-use external validation (G4), are required.
Marrow-sparing immunomonitoring (39)	n=18; historical comparator n=11; definitive CRT; treatment exposure rather than a fixed dose landmark	Lower pelvic-bone dose did not prevent lymphopenia or functional immune change	Exploratory treatment-associated immune and hematologic changes. Outcome-linked biomarker validation (G3) and locked external validation (G4) remain unestablished.
Immune-toxicity multi-omics (78)	n=30; 20 patients received CRT then anti-PD-1, and 10 received CRT then chemotherapy; 96 serial samples; on-treatment sampling at approximately 18 Gy, post-CRT/pre-systemic therapy, and during systemic treatment	T-, B- and myeloid-cell trajectories associated with immune-related adverse events	G3: immune-toxicity association/model; partial G4: independent testing in heterogeneous irAE datasets. Locked validation for the intended LACC population, treatment sequence, and landmark, and utility of earlier detection (G6), remain unestablished.
Mid-treatment MRI volume reduction (58)	n=281; two centers; 24–36 Gy; definitive radiotherapy; dose-resolved	Each 10-percentage-point higher volume reduction: OS HR 0.71; progression HR 0.74	G3: prognosis; partial G4: two-center internal-external cross-validation. Independent external validation was not feasible in the source study. Transport at the same 24–36 Gy landmark requires confirmation; the association cannot be assumed at 10–22 Gy.
MRI delta radiomics (59)	n=110; baseline and mid-treatment; CRT; time-based imaging landmark	Validation AUC 0.79 for 5-year progression-free survival	G3: prognosis with an internal holdout (37 of 110 patients). No independent external validation (G4); the delta signature did not significantly outperform the pretreatment signature (P ≥0.79).
Prospective HPV ctDNA validation (68)	70 baseline HPV-seq-positive patients; end, 4–6 weeks, 3 months; dPCR-evaluable n=62, 60, 67, respectively; definitive CRT; post-treatment landmarks	Persistent detection associated with relapse; median lead time 5.9 months	G3 with independent prospective multicenter confirmation (G4-level for the stated assay and post-treatment landmarks) in patients accrued after the pilot. Transport to other assays or populations requires separate validation.
CALLA ctDNA analysis (73)	185 baseline samples; post-treatment landmarks; randomized definitive CRT trial	Tumor-informed human-variant ctDNA detection preceded progression by median 164 days; circulating HPV DNA was assessed separately	G3: prognosis/monitoring in randomized-trial data. Trial provenance does not establish biomarker-by-treatment interaction; independent intended-use validation (G4) remains required.
CerviPro multimodal model (65)	n=1, 018; pre/post CT and clinical data; definitive radiotherapy	C-index 0.81 internal; 0.70 and 0.66 external	G3: prognosis with testing in two independent external cohorts (G4-level for the reported task).
DeepMR-LACC radioproteomics (56)	386 development; 168 internal; 93 external; proteomics n=46; pretreatment prognostic model	External C-index 0.69 for progression and 0.65 for survival; immune link in n=46	Imaging component: G3 prognosis with independent external testing (G4-level for the reported task). The 46-patient radioproteomic component provides exploratory G3 risk association/G1 immune characterization and lacks independent validation.

AUC, area under the receiver operating characteristic curve; ctDNA, circulating tumor DNA; HPV, human papillomavirus; HR, hazard ratio; MRI, magnetic resonance imaging. G1, biological plausibility; G2, analytical validity; G3, clinical association; G4, locked external validation; G5, treatment-by-biomarker interaction; G6, clinical utility. Partial G4 indicates validation with limitations for the intended assay, population or landmark.

**Table 3:** Biomarker functions and the evidence required for each claim.

Function	Question	Example in cervical chemoradiotherapy	Minimum validation requirement
Response marker	Quantifies change after treatment begins	MRI tumor-volume reduction; HPV ctDNA decline	Repeatable assay, standardized landmark, and evidence of treatment-related biological change; claims about clinical outcomes require endpoint-specific validation
Prognostic marker	Estimates outcome under a defined treatment pathway	Delta-radiomic risk; post-treatment HPV ctDNA persistence	Independent validation in the intended population and landmark; accounting for clinical covariates and transport across centers/eras; calibration when absolute risk is estimated
Treatment-predictive marker	Identifies differential benefit from one regimen relative to another	Candidate dynamic state for checkpoint timing or intensification	Prespecified treatment-by-biomarker interaction, with marker measurement before randomization for the treatment decision or a causal analysis addressing post-randomization selection; separate within-group significance is insufficient (10, 24)
Monitoring marker	Detects residual or recurrent disease at serial landmarks	HPV ctDNA at end, 4–6 weeks and 3 months	Analytical performance, standardized landmarks, confirmatory sampling where appropriate, lead time, and false-positive/false-negative consequences; clinical utility requires evidence that acting on the result improves outcomes

General immune activation denotes measurable inflammatory or cellular changes, such as interferon transcription, activation-marker expression, or cytokine release. Antitumor immune activation requires tumor-relevant evidence of recognition and functional activity. Tumor-associated clonotype expansion is supportive but does not by itself establish specific killing; CD8 density, PD-L1 induction, and generalized inflammation can also reflect tissue injury, repair, infection, or toxicity (25, 27, 38–40). The suppressive side is equally important. Pelvic fields expose circulating lymphocytes, marrow, lymphoid tissue, bowel, and tumor-draining nodes. Cisplatin and repeated fractions can reduce lymphocyte counts and function, while injured tissues release cytokines that recruit myeloid cells and promote repair. Severe radiation-induced lymphopenia was associated with worse overall and progression-free survival in a meta-analysis of eight cervical cancer cohorts (952 patients), although residual confounding and reverse causation cannot be excluded (41). In individual retrospective cohorts, profound treatment-related lymphopenia was common and associated with inferior outcomes (42–44).

Marrow sparing and immune sparing are related but not equivalent. In the INTERTECC phase II/III program, PET-guided bone-marrow-sparing intensity-modulated radiotherapy reduced acute grade 3 or higher neutropenia in the randomized component but did not reduce treatment-related lymphopenia or improve progression-free or overall survival (45). A separate randomized trial of 192 patients reduced grade 3 or higher hematological toxicity and improved cisplatin completion with active-marrow-sparing volumetric-modulated arc therapy, without a demonstrated survival advantage (46). Across randomized trials with mixed definitive and postoperative populations, marrow sparing reduces hematological toxicity and chemotherapy interruption, but survival data remain insufficient (47). Direct longitudinal immunomonitoring adds a caution: in 18 women treated with marrow-sparing volumetric-modulated arc therapy, a mean pelvic-bone-dose reduction of 8.1 Gy did not prevent leukopenia, lymphopenia, loss of CD4 T and B cells, or reduced pathogen-specific T-cell reactivity; comparison with 11 historically treated patients was exploratory (39).

The strongest argument for dynamic biomarkers comes from direct serial sampling (**Figure 1**). In 20 patients undergoing chemoradiotherapy, minimally invasive cervical cytobrushing at baseline and weeks 1, 3, and 5 showed an early decline in CD4 and CD8 T cells, followed by variable expansion. Proliferating Ki67-positive CD8 T cells peaked around week 3, while CD69 expression increased across treatment. Several local changes were absent from matched blood (40). This temporal pattern argues against labeling a single mid-treatment sample as “inflamed” or “suppressed” without knowing where it lies on the trajectory.

**Figure 1:**
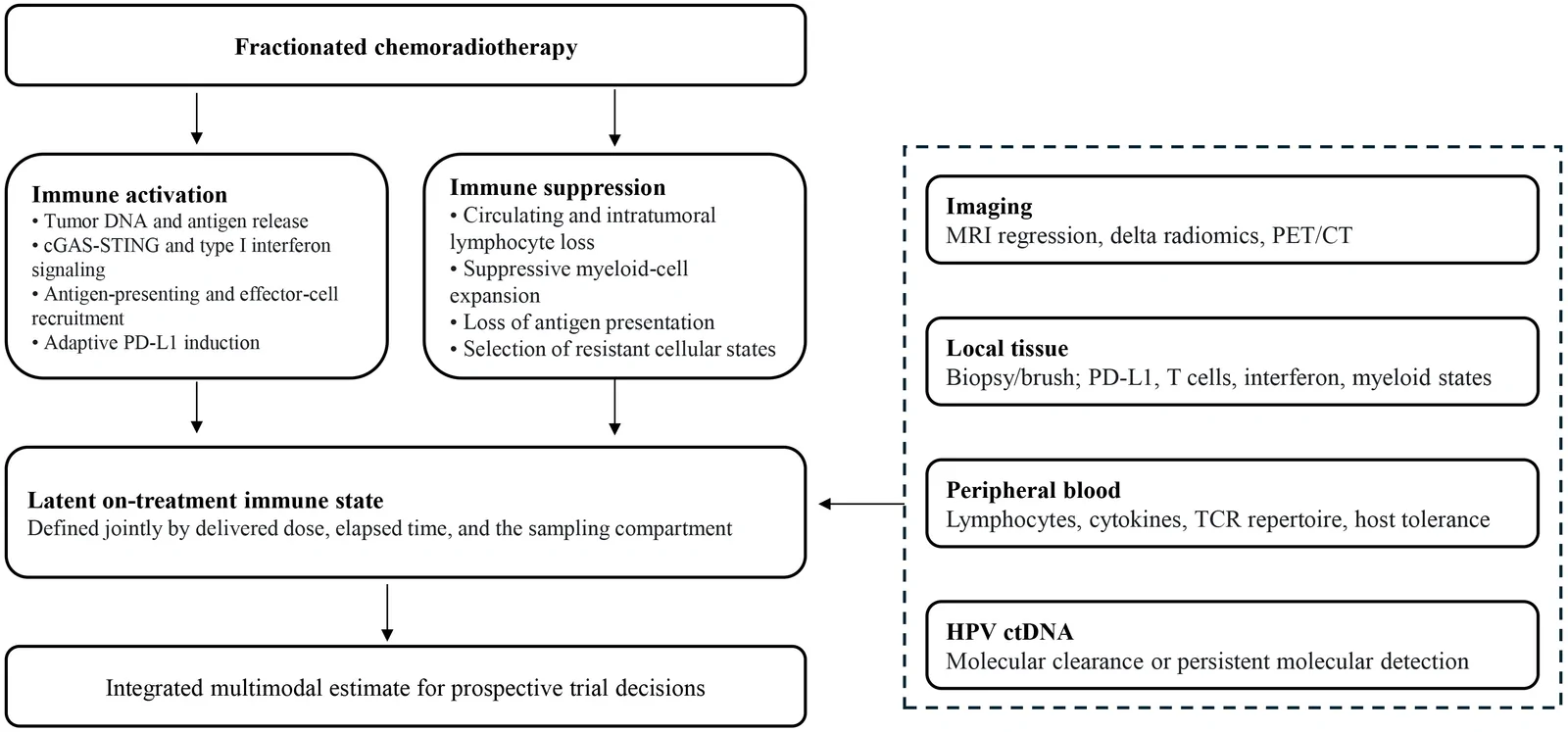
Bidirectional immune effects of fractionated chemoradiotherapy and their observable surrogates. Radiation and cisplatin can simultaneously activate antigen release, cytosolic-DNA sensing, interferon signaling and effector-cell recruitment while depleting lymphocytes, promoting suppressive myeloid states, reducing antigen presentation and selecting resistant cells. MRI/PET, local tissue, peripheral blood and HPV circulating tumor DNA observe complementary projections of a latent, dose- and time-dependent state. cGAS, cyclic GMP–AMP synthase; HPV, human papillomavirus; MRI, magnetic resonance imaging; PD-L1, programmed death-ligand 1; PET/CT, positron-emission tomography/computed tomography; STING, stimulator of interferon genes.

A prospective cohort of 55 patients sampled tumor and blood before, during, and after chemoradiotherapy. CD4 and PD-1-positive circulating T cells decreased, regulatory T cells increased, intratumoral CD4 and CD8 T cells declined, and median tumor PD-L1 positivity fell. Greater pretreatment peripheral T-cell receptor diversity was associated with better progression-free and overall survival (48). In a separate paired-biopsy study, however, immune response after 20 Gy was markedly heterogeneous: CD8 infiltration increased in some tumors and decreased in others. Increased CD8 infiltration was linked to nuclear IRF1 and short-term response, and PD-L1 increased in approximately 40% of tumors, particularly when peripheral interferon signatures rose (49). Sampling at other times produces different pictures. Paired pretreatment biopsies and post-radiation resection specimens from 104 patients treated with preoperative radiotherapy with or without chemotherapy showed increased tumor-cell PD-L1 and decreased CD8 and FOXP3 infiltration after chemoradiotherapy; post-treatment tumor-cell PD-L1 positivity was associated with better survival and less out-of-field recurrence (50). In a prospective study of 19 patients sampled before chemoradiotherapy, before brachytherapy, and two weeks after completion of brachytherapy, PD-L1 expression at the final sampling point was higher in the high-dose-rate than the pulsed-dose-rate cohort; equipment-dependent allocation and the small, nonrandomized comparison limit inference (51). At treatment completion in a prospective cohort of 22 patients, intratumoral T cells and tumor PD-L1/MHC-I were reduced while macrophages and neutrophils increased (52). More recent dose-anchored sampling adds another layer. Among 40 patients, PD-L1 was commonly upregulated after 10–14 Gy and 18–22 Gy, accompanied by NF-κB activation and increased CD8 T-cell and macrophage infiltration (53). A 2026 multicohort analysis integrating bulk and single-cell transcriptomics mapped extensive chemoradiation-induced reprogramming and identified MDM2 as a candidate resistance node; MDM2 inhibition improved radiation response and altered the immune landscape in preclinical HPV-positive models (54).

Reanalysis of publicly available single-cell data from paired specimens of three patients, obtained one week before and three weeks after initiation of chemoradiotherapy, identified a candidate suppressive macrophage circuit:1 early on-treatment tumor cells showed higher CD24 and MUC16 expression, while macrophages expressed more Siglec-10, Siglec-9, and PD-L2; functional analyses supported impaired tumor-cell elimination and T-cell inhibition (55). The study provides a plausible resistance mechanism, but it does not establish that blocking this circuit improves outcomes during definitive therapy. This distinction—mechanistic target versus clinically validated adaptive action—applies equally to MDM2, suppressive macrophage states, and checkpoint induction. The apparent contradictions are clinically productive. Early interferon-driven PD-L1 induction may define a transient checkpoint-sensitive window, whereas later lymphocyte depletion, loss of antigen presentation, or myeloid dominance may indicate a closing window.

## Dynamic biomarkers across imaging, molecular, and immune compartments

### Imaging trajectories and multimodal models

AI is an umbrella term; machine learning denotes models learned from data, and deep learning is its neural-network subset. Radiomics denotes quantitative image-feature extraction, delta radiomics longitudinal feature change, multimodal fusion the combination of distinct data modalities, and radioproteomics the relation of imaging to proteomic measurements (20–23, 56, 57).

MRI is already embedded in cervical radiotherapy and brachytherapy workflows, making it the most practical platform for repeated tumor assessment. Tumor volume reduction is interpretable, inexpensive relative to omics, and less vulnerable than high-dimensional radiomics to feature instability. In a two-center retrospective study of 281 patients with pre- and mid-treatment MRI obtained at 24–36 Gy, each 10-percentage-point increase in tumor volume reduction rate was associated with better overall survival (HR, 0.71), progression-free survival (HR, 0.74), and local control (subdistribution HR, 0.68). Two-center internal-external validation yielded C-statistics of 0.72-0.79 with acceptable 60-month calibration (58). Modeling reduction continuously rather than dichotomizing at an arbitrary cutoff is a notable strength and provides a useful design variable for trials. The prognostic association shown at 24–36 Gy cannot be assumed to apply at the proposed earlier 10–22 Gy windows without separate validation.

Delta radiomics aims to capture biological change beyond size. In 110 patients, a T2-weighted MRI delta signature derived from baseline and mid-treatment scans predicted 5-year progression-free survival with an area under the time-dependent receiver-operating-characteristic curve (AUC) of 0.79 in the validation cohort, compared with 0.75 for a pretreatment signature; performance did not differ significantly between the signatures in either cohort (P ≥0.79) (59). In another cohort of 111 patients, models based on change from baseline to pre-brachytherapy MRI produced test-set AUCs of 0.759 for 2-year progression-free survival and 0.789 for overall survival; adding clinical variables increased AUCs to 0.812 and 0.868 (60). These numerical gains are encouraging but modest relative to the additional acquisition, segmentation, harmonization, and missing-data burden. Static MRI models remain relevant as a benchmark. An international multicenter study trained a T2-weighted MRI radiomic signature in 132 patients and validated it in an independent 199-patient cohort. The signature remained associated with disease-free survival after adjustment for stage and tumor volume, but the adjusted validation HR was 1.84, smaller than in training (61).

Deep learning can integrate multiple images and reduce handcrafted feature dependence, but it does not remove design bias. A 2.5-dimensional multisequence MRI model for complete response in 259 patients from two centers achieved a test AUC of 0.814; adding clinical variables increased it to 0.839 (62). The endpoint was post-treatment response, not benefit from an alternative therapy. A scoping review of 153 cervical cancer machine-learning studies, including deep learning, found that diagnosis dominated the literature and that reproducibility remained a major obstacle (63). A systematic review of 133 artificial-intelligence studies in gynecologic oncology found that 76% were single-center, 94% retrospective, and 95% enrolled fewer than 500 patients. Among the 81 studies predicting treatment response, prognosis, or adverse events, only three (4%) used temporal-related unconventional radiomics (64). Dynamic imaging is therefore conceptually attractive but still empirically sparse.

Large multicenter models illustrate both the value and limits of fusion. CerviPro integrated pre- and post-treatment CT, handcrafted radiomics, and clinical variables in 1, 018 patients receiving definitive radiotherapy. The model achieved a C-index of 0.81 internally but 0.70 and 0.66 in two external cohorts (65). The external decline is more informative than the internal result: it quantifies the cost of transport across centers and potentially across scanners, treatment eras, populations, and follow-up practices. DeepMR-LACC combined pretreatment T2-weighted MRI with an automated multitask model and investigated radioproteomic associations. The imaging model was developed in 386 patients and tested internally in 168 and externally in 93; external C-indices were 0.69 for progression-free and 0.65 for overall survival. Proteomic profiling linked the imaging-defined high-risk group to an immunosuppressive microenvironment, and a radioproteomic model reached C-indices of 0.85 for both endpoints in a 46-patient cohort (56). The biological link is important, but the small radioproteomic cohort makes the higher performance vulnerable to overfitting.

Direct correlations between imaging and immune assays are generally modest. In 89 patients, selected MRI radiomic features correlated with PD-L1 combined positive score, proliferation, stromal composition, and vimentin; the strongest reported correlation with PD-L1 was approximately -0.38 (66). A PET/CT radiomics study analyzed separate subsets for segmentation, PD-L1 association, and immunotherapy response and reported an AUC of 0.935 for a support-vector-machine response model in 85 patients (67). The most defensible near-term role for multimodal artificial intelligence is joint prognostic and monitoring support: integrating baseline burden, early change, residual molecular disease, and host immune tolerance to estimate absolute risk. Treatment selection should be a separate, prospectively tested layer. A recent narrative review of radiomics and artificial intelligence in cervical precision radiotherapy reached a similar translational conclusion: encouraging technical performance has not yet established clinical impact (16).

### Molecular residual disease, host immunity, and toxicity

HPV ctDNA has a distinct advantage over many imaging markers: it is tumor-linked, serially measurable, and biologically specific for most cervical cancers. In a prospective multicenter validation cohort, 70 patients had detectable HPV ctDNA by HPV-seq at baseline. Detectable HPV ctDNA at the end of chemoradiotherapy, 4–6 weeks after treatment, and three months after treatment was associated with inferior two-year progression-free survival. On dPCR, two-year progression-free survival was 82% versus 15% at 4–6 weeks (n=60) and 82% versus 24% at three months (n=67), comparing undetectable with detectable HPV ctDNA; median lead time to recurrence was 5.9 months (68). Smaller studies using pan-HPV sequencing or multi-genotype assays similarly showed that persistent or rising circulating HPV DNA tracked incomplete response or relapse (69, 70). A 2026 cohort with 31 enrolled patients (27 analyzed) reported that on-treatment plasma HPV ctDNA negativity was independently associated with favorable early treatment response in an exploratory multivariable analysis, but its size and single-cohort design require validation (71). Separately, an August 2026 retrospective safety-net pilot of 17 patients using a tumor-informed human-variant assay further showed why trajectories matter: all five recurrences occurred among seven patients with persistent or recurrently positive tumor-informed ctDNA, whereas none occurred among eight patients with sustained clearance, and isolated negative measurements appeared within otherwise positive courses (72).

The CALLA translational analysis provides a substantial randomized-trial biomarker dataset among the studies reviewed here. A tumor-informed ultrasensitive assay analyzed up to 1, 800 patient-specific human variants in 185 baseline samples, with repeat measurements after chemoradiotherapy and at three months; circulating HPV DNA was assessed separately. Baseline ctDNA was detectable in 98.9% of samples. Post-treatment detection preceded clinical progression by a median of 164 days, and ctDNA/circulating HPV DNA levels were associated with progression and survival (73).

Genotype-matched digital PCR, broad HPV-targeted sequencing, and tumor-informed human-variant assays differ in target coverage, analytical sensitivity, quantification, and tissue requirements (68–70, 73). Human-variant ctDNA and circulating HPV DNA are related but analytically distinct measurements. An actionable molecular-residual-disease strategy requires more than analytical sensitivity. False reassurance can occur when a tumor sheds little DNA, the relevant HPV genotype is not captured, plasma volume is insufficient, or clearance reflects local control while occult non-shedding disease persists. A negative HPV ctDNA result is also less informative in HPV-independent disease or when baseline detectability has not been established. Preanalytical handling and sampling time can further affect apparent performance. Conversely, transient low-level detection may not warrant treatment escalation.

Peripheral blood is repeatable and scalable, whereas tissue is spatially specific and invasive. Serial blood studies show substantial and prolonged loss of circulating T cells and impaired antigen-specific or mitogenic responses during pelvic chemoradiotherapy, with recovery over subsequent weeks (74). Baseline and post-treatment lymphocyte counts may index host immune reserve, radiation exposure to circulating cells, marrow dose, inflammation, nutritional status, infection, and tumor burden simultaneously. They are therefore accessible but nonspecific. Tissue assays resolve local immune states but face sampling error as tumors shrink and become necrotic. Baseline tumor-infiltrating lymphocytes have been associated with outcome after chemoradiotherapy (75). In a preoperative cohort of 38 patients receiving (chemo)radiotherapy followed by surgery, tissue CD8, macrophage, IL-33, and PD-L1 markers and their changes were associated with pathological response or cause-specific survival, with directions varying across markers; these findings are indirect for definitive chemoradiotherapy (76). Bulk RNA sequencing of 20 paired tumor samples collected before and after radiotherapy suggested increased immune infiltration and an exploratory association with short-term response; the small sample warrants independent validation before transport (77). Here, immune competence means the host capacity to maintain antigen presentation and functional immune-cell responses during treatment. It is a multidimensional research construct, not a validated score or a synonym for absolute lymphocyte count. Blood counts, immune-cell composition, functional assays, and infection or immunosuppression history provide different, incomplete observations (39, 74).

A pragmatic platform should use blood at every decision window and reserve tissue for baseline and one carefully selected on-treatment time point, such as after approximately 20 Gy, when several studies observed interferon signaling, PD-L1 induction, or emerging heterogeneity. Minimally invasive cervical brushing may increase feasibility but samples surface-accessible compartments and requires assay-specific validation. Imaging can provide whole-tumor and whole-body context, while ctDNA supplies a systemic residual-disease signal. The modalities are complementary precisely because none is complete. An efficacy-oriented state model should not absorb immune-related adverse events into a generic “unfavorable” category. Longitudinal single-cell multi-omics in patients receiving chemoradiotherapy followed by anti-PD-1 therapy linked immune-related adverse events to expansion of terminally differentiated effector T-cell populations, altered B-cell clonality and class switching, specific monocyte states, and spatial aggregation of these cells in the tumor microenvironment (78). The irAE models were tested in independent datasets across heterogeneous populations and sample types. This external evidence does not establish locked validation in the intended LACC population at the stated sampling landmark, or clinical utility of earlier detection without compromising anticancer therapy. Efficacy, treatment-related lymphopenia, hematological toxicity, and immune-related adverse events therefore require distinct endpoints and competing-risk-aware analyses.

## From association to adaptive decisions

We propose five clinical windows (**Figure 2**). At baseline, the objective is to establish disease extent, radiotherapy quality requirements, HPV genotype, immune competence, and eligibility for the evidence-based checkpoint regimen. During early external-beam radiotherapy, at reported dose landmarks such as 10–14 and 18–22 Gy, with elapsed days recorded separately, the objective is to measure immune activation versus suppression before profound lymphocyte loss or resistant-state selection dominates. At pre-brachytherapy imaging, tumor regression and anatomy inform brachytherapy while dynamic imaging and blood markers update residual-risk estimates. At treatment completion and 4–6 weeks, HPV ctDNA clearance and recovery of host immunity can inform assessment of molecular residual risk, with assay-specific confirmation. At three months, integrated imaging and ctDNA are proposed to distinguish durable response, inflammatory change, and persistent disease, with confirmatory clinical assessment.

**Figure 2:**
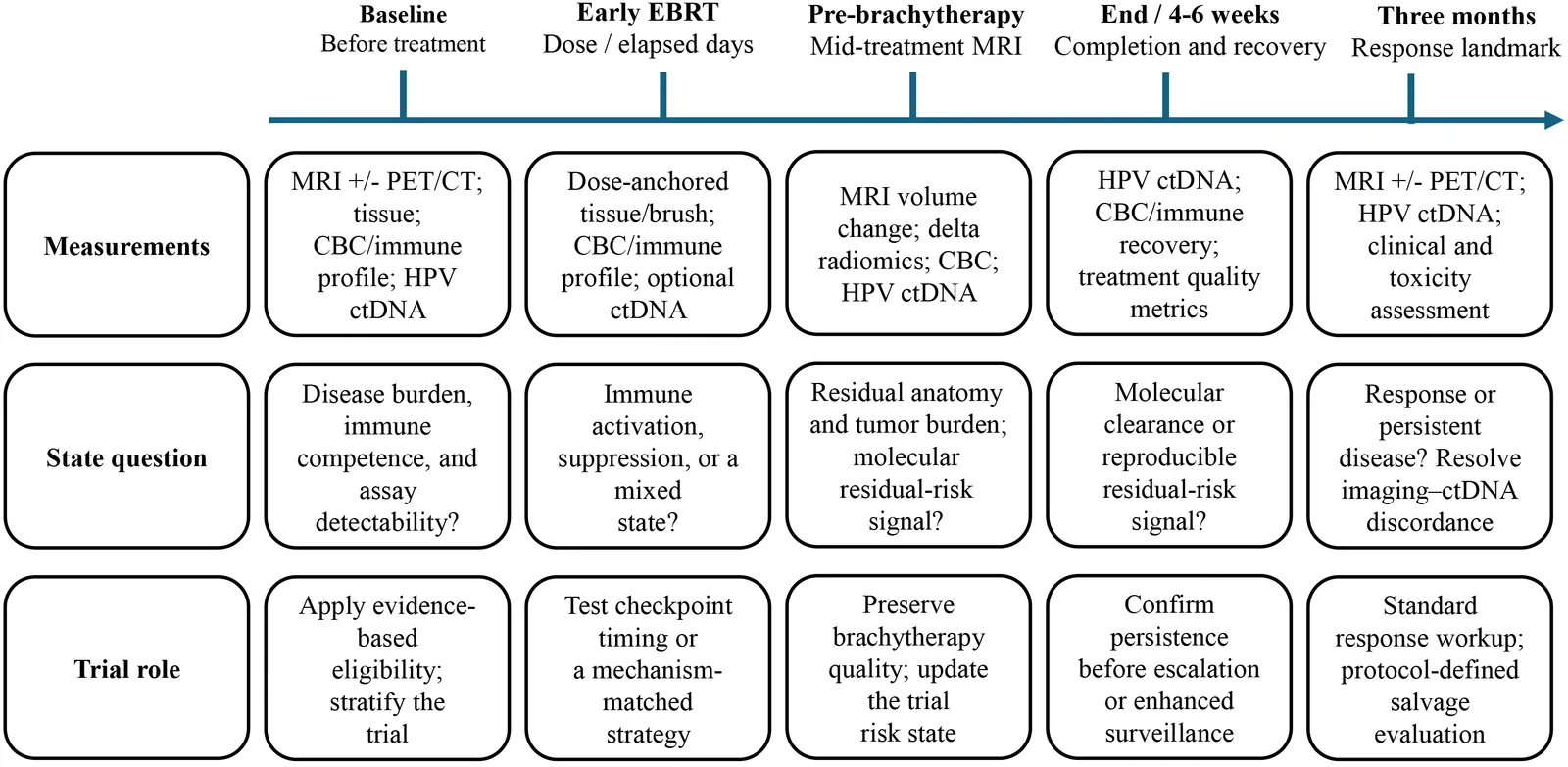
Proposed decision-anchored sampling framework for adaptive cervical immunoradiotherapy trials. Each window answers a distinct state question and maps to a prospective trial action. CBC, complete blood count; ctDNA, circulating tumor DNA; EBRT, external-beam radiotherapy; HPV, human papillomavirus; MRI, magnetic resonance imaging; PET/CT, positron-emission tomography/computed tomography.

Each window must map to a feasible action. Potential trial actions include timing or continuation of checkpoint blockade, enrollment into a resistance-targeted strategy, lymphocyte-sparing radiotherapy optimization, intensified surveillance, or early salvage evaluation. The proposed windows should first undergo a prospective feasibility run-in to measure sampling success, assay availability, and turnaround against a fixed decision deadline. The marker or state should then undergo locked external validation, followed by randomized evaluation of the proposed action and its clinical utility.

Repeated measurements are clustered within patients and are affected by informative missingness: patients with rapid progression, toxicity, poor imaging quality, or inaccessible residual tumor are more likely to miss later samples. Treating all time points as independent observations exaggerates precision. Depending on the estimand, appropriate approaches include mixed-effects models, joint longitudinal-survival models, landmark analysis, multi-state models, and time-dependent Cox models. Center and era are not nuisance variables to be randomly dispersed across folds. Scanner manufacturer, field strength, reconstruction, segmentation, brachytherapy technique, contouring, checkpoint availability, and supportive care can all encode center or calendar time. A credible validation hierarchy is patient-level internal resampling, temporally separated validation, internal-external validation by center, and fully external validation. The CerviPro and multicenter MRI studies show why this hierarchy matters: CerviPro showed lower external discrimination, while the MRI signature showed attenuated adjusted prognostic associations in its external cohort (61, 65).

Calibration is essential because adaptive decisions depend on absolute risk, not rank alone. A model with an acceptable C-index can substantially overestimate risk in a new center or treatment era. Calibration-in-the-large, calibration slope, time-specific calibration plots, and uncertainty intervals should be reported. Decision-curve analysis can estimate whether a rule offers net benefit across plausible thresholds, but it cannot repair bias, substitute for a randomized utility trial, or determine whether the proposed intervention is effective. TRIPOD+AI, PROBAST+AI, CLAIM, Image Biomarker Standardisation Initiative recommendations, and the readiness-level logic of Radiomics Quality Score 2.0 should be specified prospectively (20–23, 57).

## A prospective roadmap for dynamic multimodal immunoradiotherapy

**Table 4** summarizes the translational pathway. The first stage should be a multicenter prospective cohort embedded in standardized definitive chemoradiotherapy, with treatment and sample collection completed without biomarker-directed changes. A minimum core dataset would include baseline and pre-brachytherapy pelvic MRI, radiotherapy dose and timing, weekly complete blood counts, baseline and serial plasma for HPV ctDNA, and standardized outcomes. An enhanced cohort would add blood immunophenotyping, cytokines, T-cell receptor sequencing, and optional tumor tissue at baseline and approximately 20 Gy. Collection should be anchored to delivered dose and elapsed time, because “mid-treatment” can represent biologically different exposure.

**Table 4:** Translational pathway from dynamic association to an adaptive clinical decision.

Stage	Sampling window	Core inputs	Primary evidentiary goal	Guardrail
1. Prospective observation	Baseline; 10–22 Gy with elapsed days recorded separately; pre-brachytherapy; end; 4–6 weeks; 3 months	MRI, blood counts, HPV ctDNA; enhanced tissue and immune assays	Incremental prognostic/monitoring value beyond clinical and treatment-quality variables	No biomarker-directed treatment change; assess assay availability, sampling failures and turnaround before a fixed decision deadline
2. Locked external validation	Same prespecified landmarks	Frozen preprocessing, state definitions and thresholds	Calibration, discrimination, decision curves, missing-modality performance, subgroup transport	Center- and era-separated testing; fallback model
3. Randomized adaptive utility	Single actionable landmark chosen before enrollment	Validated state plus mechanism-matched intervention	Treatment effect and intent-to-treat clinical utility at the selected landmark; interaction across states requires comparative treatment data in both states	Preserve chemoradiotherapy and brachytherapy quality; no delay while awaiting research results
4. Routine clinical use	Real-time acquisition and monitored deployment	Versioned assay/model with uncertainty and out-of-distribution checks	Outcome, toxicity, failures, drift and equity under routine use	No silent updating; recalibrate or revalidate after material change

The primary estimand should be clinically explicit, for example: the incremental ability of pre-brachytherapy MRI change plus contemporaneous HPV ctDNA to estimate subsequent three-year progression-free survival among patients eligible at that landmark beyond stage, nodal status, tumor volume, histology, and treatment quality. Secondary estimands can address local failure, distant failure, overall survival, immune-related adverse events, and severe lymphopenia. Competing risks are appropriate for site-specific failure, and noncancer death should not be silently treated as censoring when relevant.

The data model should preserve raw timestamps, radiation dose at collection, assay version, failure codes, and reasons for missingness. Imaging preprocessing, feature definitions, and segmentation should comply with image-biomarker standards. Model development should anticipate absent modalities: complete-case multimodal fusion is likely to select healthier patients treated at better-resourced centers. Modality dropout during training, missingness indicators, multiple imputation appropriate to the analysis model, and a validated fallback model are preferable to discarding patients. The output should be a small number of interpretable states rather than an opaque probability at every fraction. One plausible state taxonomy is: favorable early regression with clearance of a baseline-detectable molecular signal; favorable regression with persistent molecular detection; poor regression with immune activation; poor regression with immune suppression; and indeterminate because of missing or discordant data. The taxonomy is a hypothesis and must be learned and validated, not imposed as biological truth. **Table 5** sets out the biological hypothesis, alternative explanations, and confirmation evidence required for each candidate combination. External validation should freeze preprocessing, feature definitions, coefficients, and decision thresholds. Performance should be reported by center, age, stage, nodal status, histology, HPV genotype, geographic region, and radiotherapy technique, with confidence intervals. Fairness assessment should focus on clinically plausible mechanisms of error, including differential access to serial MRI or high-sensitivity molecular testing.

**Table 5:** Candidate immune-state hypotheses, alternative explanations, and confirmation requirements.

Candidate observations	Biological hypothesis	Alternative explanations	Evidence required
Early regression with clearance of a baseline-detectable molecular signal	Treatment response with declining molecular burden	Limited assay sensitivity, changed shedding, or occult non-shedding disease	Baseline detectability; repeat standardized sampling; clinical/imaging correlation and external outcome validation
Regression with persistent molecular detection	Residual viable disease despite anatomical response	Delayed clearance, transient DNA release, altered shedding, or analytical variation	Serial confirmation; assay/HPV coverage; longitudinal outcomes and clinical or pathological adjudication where appropriate
Poor regression with immune activation	Candidate active immune response without prompt anatomical regression	Inflammation, tissue repair, delayed response, or ineffective immune activation	Tumor-relevant functional evidence; serial imaging and molecular assessment; outcome-linked external validation
Poor regression with immune suppression	Candidate impaired antitumor immune response	Treatment-related lymphopenia, compartment mismatch, sampling failure, or nonimmune causes of slow regression	Paired local/systemic assays; standardized sampling; mechanism-specific and outcome-linked confirmation
Missing or discordant modalities	No reliable state assignment	Assay failure, different biological compartments or sampling times, or absent baseline detectability	Record missingness; repeat/adjudicate measurements; validate fallback management and retain an indeterminate category

Only the third stage can support treatment selection. Patients would receive standard chemoradiotherapy and evidence-based checkpoint blockade according to current eligibility. At a prespecified landmark, those in a high-risk dynamic state could be randomized to standard continuation versus a biologically matched strategy, while favorable-state patients continue standard treatment. The intervention might address a defined mechanism—persistent checkpoint-active inflammation, molecular residual disease, suppressive myeloid remodeling, or a target such as MDM2—rather than generic “intensification.” This high-risk-only randomization estimates the treatment effect within that state and cannot by itself test treatment-by-state interaction across high-risk and favorable states; interaction validation would require comparative treatment data in both states. The trial should preserve intent-to-treat comparisons and account for landmark eligibility. Clinical utility includes survival, toxicity, patient-reported outcomes, treatment completion, and consequences of false-positive and false-negative assignments. A biomarker is useful only if the action it triggers improves outcomes enough to justify assay burden and treatment harm. If utility is established, implementation requires quality control beyond model discrimination. Sites need acquisition tolerances, assay turnaround targets, automatic detection of out-of-distribution inputs, auditable versioning, and a multidisciplinary review process. Predictions should display the contributing time points, uncertainty, calibration date, and valid population. Changes in scanners, contouring, brachytherapy, HPV assays, or checkpoint regimens require documented impact assessment and, when necessary, recalibration or revalidation. **Table 4** summarizes this pathway. The central design principle is that the biomarker should be developed around a decision and a time window.

## Discussion

The immunotherapy era changes what counts as a useful biomarker in LACC. Before KEYNOTE-A18, many models reasonably focused on recurrence risk after chemoradiotherapy. Now, a clinically decisive biomarker must address at least one of three questions: whether the checkpoint-containing regimen is producing its intended biological effect, whether residual disease is emerging despite that effect, or whether a different strategy offers greater net benefit. Static baseline models remain useful for stratification, but they cannot answer these questions alone.

Three conclusions are supported by the current evidence. First, cervical tumors undergo measurable immune remodeling during treatment, and the direction is heterogeneous. Serial human studies repeatedly show changes in CD8 T cells, PD-L1, interferon signaling, T-cell receptor diversity, macrophages, and systemic lymphocytes, but the result depends on dose and sampling time (40, 48, 49, 53). This heterogeneity supports, rather than undermines, time-resolved sampling. Second, dynamic tumor burden carries reproducible prognostic information. Mid-treatment MRI volume reduction shows a strong continuous association with outcome, and delta-radiomic models show potential prognostic value beyond stage or baseline size, although comparative gains are not consistently significant (58–60). Third, among the molecular monitoring approaches reviewed here, post-treatment HPV ctDNA currently has some of the strongest prospective validation for residual-risk assessment and monitoring (68). The CALLA analysis provides complementary prognostic and monitoring evidence for tumor-informed human-variant ctDNA and separately assessed circulating HPV DNA (73).

Safety is a parallel dynamic objective rather than a secondary annotation. Longitudinal multi-omics demonstrates that immune-related adverse events have measurable cellular trajectories, while marrow-sparing trials show that reducing hematological toxicity does not automatically preserve lymphocytes or establish better tumor control (39, 45, 78). A clinically useful adaptive platform must therefore model therapeutic benefit, immune toxicity, chemotherapy delivery, and false treatment escalation separately. The major gap among the studies reviewed is the absence of validated differential checkpoint-treatment benefit identified by a prespecified dynamic marker. PD-L1 is biologically dynamic and assay-dependent; imaging-immune correlations are modest; ctDNA identifies residual risk but not necessarily the therapy that removes it; and multimodal models are typically developed in patients receiving one treatment pathway. Claims should therefore be calibrated to study design. The field needs fewer reports that relabel prognosis as “immunotherapy efficacy” and more randomized interaction analyses with prespecified assays.

Several controversies deserve direct testing. The first is checkpoint timing. Early PD-L1 induction and T-cell activation suggest a window during fractionated radiation, while lymphocyte depletion and late antigen-presentation loss suggest that the window can close. NRG-GY017 and COLIBRI provide proof that neoadjuvant therapy changes immune measurements, but not definitive comparative efficacy (27, 28). The second is local-systemic discordance. A blood-only assay may miss tumor-local activation, whereas a single biopsy misses spatial heterogeneity and distant disease. The third is whether complex multimodal fusion adds enough information beyond MRI volume reduction plus HPV ctDNA to justify cost and missingness.

This Review has limitations. Literature selection was purposive and potentially incomplete. The six evidence gates were applied as a conceptual appraisal framework and should not be interpreted as a validated scoring system. Many dynamic immune cohorts are small and use nonidentical radiation schedules, sampling methods, and assays. AI studies often lack prospective validation, and their reported metrics cannot be compared directly across endpoints and partitions. The proposed state model also does not yet incorporate gut or vaginal microbiome trajectories. A newly published paired study of 33 patients found radiation-associated compositional changes across tumor, vaginal, gut, urinary, and oral sites, while a contemporaneous review linked microbial states to immunity, response, and toxicity; neither establishes a validated treatment-selection rule (17, 79). These limitations define practical priorities. Prospective cohorts should pair dose-anchored local and systemic sampling with rigorous radiotherapy quality assurance. Phase 3 datasets should be used for treatment-by-biomarker interaction analyses, with attention to multiplicity, assay prespecification, and post-randomization selection when evaluating on-treatment markers (24). Dynamic models should report calibration, missingness, external performance, and failure modes. Trials should test one actionable state and one biologically matched intervention at a time.

## Conclusion

The reviewed evidence supports dose- and time-resolved biomarkers primarily for biological response assessment, prognosis, and monitoring, with insufficient validation for checkpoint-regimen selection. Treatment selection requires a prespecified, reproducible biomarker state measured at a defined landmark and validated to identify differential benefit from alternative checkpoint regimens. Biomarker-guided checkpoint changes should remain within prospective trials until they are shown to improve patient outcomes without compromising definitive chemoradiotherapy or brachytherapy.
